# Pulmonary Phaeohyphomycosis Caused by *Phaeoacremonium* in a Kidney Transplant Recipient: Successful Treatment with Posaconazole

**DOI:** 10.1155/2014/902818

**Published:** 2014-05-14

**Authors:** Saivaralaxmi Monaganti, Carlos A. Q. Santos, Andrea Markwardt, Morgan A. Pence, Daniel C. Brennan

**Affiliations:** ^1^The Department of Medicine, Washington University School of Medicine, 660 S. Euclid Avenue, Renal Division/Campus Box 8126, St. Louis, MO 63110, USA; ^2^Barnes-Jewish Hospital, Center for Outpatient Health, 4901 Forest Park Avenue, 5th Floor, St. Louis, MO 63108, USA; ^3^Department of Pathology & Immunology, Washington University School of Medicine, 660 S. Euclid Avenue, Campus Box 8118, St. Louis, MO 63110, USA

## Abstract

We report a rare case of pulmonary phaeohyphomycosis in a 49-year-old woman 6 years after kidney transplantation. She presented with dyspnea, cough, and fatigue. Her chest CT scan revealed nodular opacities in the right upper lung. A fine needle aspirate biopsy culture yielded *Phaeoacremonium* and surgical pathology of the biopsy showed chronic inflammation. We successfully treated her with posaconazole and managed drug interactions between posaconazole and tacrolimus. This is the second reported case of biopsy-proven pulmonary infection by *Phaeoacremonium* in a kidney transplant recipient and successfully treated with posaconazole.

## 1. Background

*Phaeoacremonium* species are well known plant pathogens causing stunted growth and dieback of various woody hosts especially grapevines and have been isolated from necrotic woody tissue of Prunus species [1, 2].* Phaeoacremonium* species are dematiaceous fungi characterized by the presence of melanin or melanin-like pigments and are widely distributed in the environment particularly in soil, wood, and decomposing plant debris. Phaeohyphomycosis is a collective term for cutaneous, subcutaneous, and systemic disease caused by dematiaceous fungi. Pulmonary phaeohyphomycosis is a rare opportunistic infection of immunocompromised hosts. A review of 34 cases of dematiaceous fungal infections in organ transplant recipients revealed an overall mortality of 57% among patients with systemic disease and 7% among those with skin, soft-tissue, or joint infections [3].

This is only the second case of biopsy-proven pulmonary infection by* Phaeoacremonium* in a kidney transplant recipient and the first report of successful treatment with posaconazole. Moreover, management of drug interactions between posaconazole and tacrolimus was successfully done, thereby preventing supratherapeutic levels of tacrolimus and avoiding kidney injury.

## 2. Case Report

**Figure 1:**
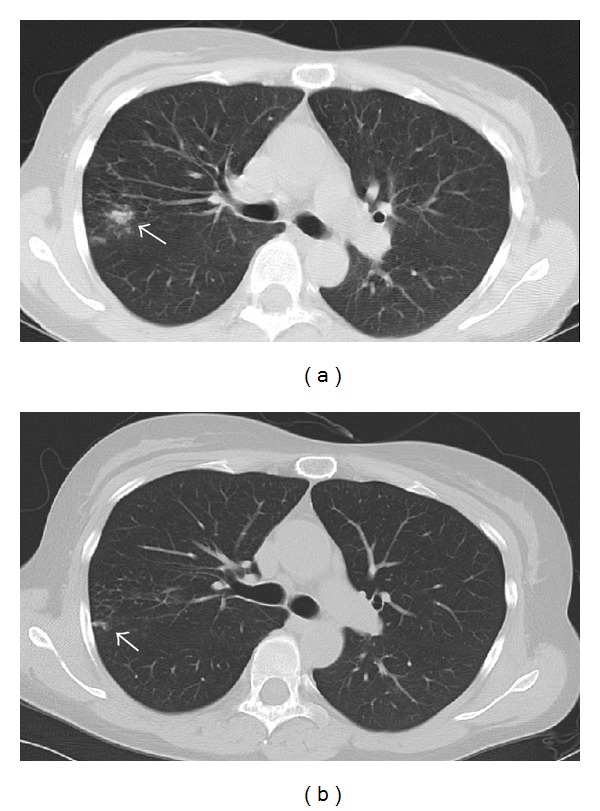
(a) Chest CT scan before starting posaconazole showing reticulonodular opacities in the right upper lobe. (b) CT scan one month after starting posaconazole showing resolution of most of the opacities.

A 49-year-old Caucasian female who underwent a living related kidney transplant 6 years before presented with progressive dyspnea, cough, and fatigue over 6 months that failed to improve after the administration of several antibiotic courses. She had been on tacrolimus and prednisone for maintenance immunosuppression. She lived in a rural area, had exposure to chicken sheds and barns, and was a gardener. A chest CT scan revealed nodular opacities in the right upper lobe (Figure 1(a)), and she underwent bronchoscopy with bronchoalveolar lavage and transbronchial fine needle aspiration biopsy of the right upper lobe nodules.

*Phaeoacremonium *species grew from the biopsy culture within four days of incubation. Identification was assigned based on macroscopic and microscopic morphology. Initially, the surface of the mold was olive in color, becoming greyish-black upon subculture. The texture was velvety, and the reverse was black. Microscopically, pigmented hyphae with tapering, funnel-shaped phialides were observed, and conidia were hyaline and oblong, forming clusters at the tip of the phialides. Macroscopic and microscopic morphology was consistent with* Phaeoacremonium* species. Surgical pathology of the biopsy showed chronic inflammation but no fungal hyphae. Culture for acid fast bacilli from the biopsy specimen was negative for mycobacteria. Culture of bronchial fluid yielded* Dactylaria constricta* and few* Mycobacterium avium-intracellulare* complex.

**Figure 2:**
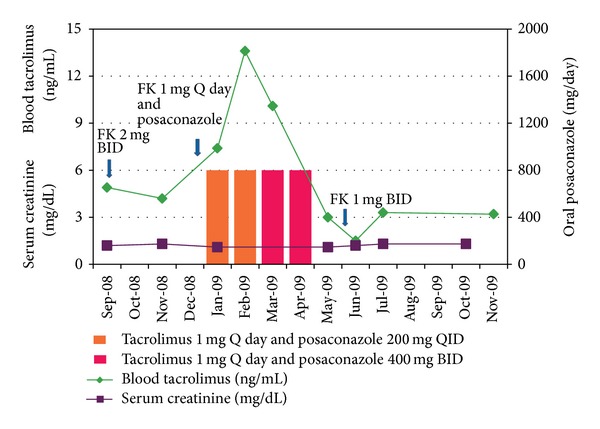
Graph showing serum creatinine and blood tacrolimus levels before, during, and after posaconazole treatment with tacrolimus dose adjustments.

Given that her biopsy culture yielded* Phaeoacremonium* and showed chronic inflammation, we started oral posaconazole 200 mg QID and reduced her tacrolimus dose from 2 mg BID to 1 mg Q day. A repeat chest CT scan one month after the institution of antifungal therapy showed improvement (Figure 1(b)), and she reported reduced cough and shortness of breath. Two months after commencing treatment, we changed her posaconazole dose to 400 mg BID for greater ease of administration. She received posaconazole for 4 months and her symptoms resolved (Figure 2).

## 3. Discussion

To et al. reported the first case of biopsy-proven* Phaeoacremonium parasiticum* lung infection in a kidney transplant recipient. In contrast to our case, the patient was severely immunocompromised due to chemotherapy for posttransplant lymphoproliferative disease. He showed initial improvement with voriconazole and caspofungin but succumbed after a prolonged period of neutropenic fever. Shah et al. described a case of probable* Phaeoacremonium* lung infection in a lung transplant recipient. The patient developed cavitary lung nodules in the native lung a few months after single lung transplantation. Biopsy of one of the nodules showed chronic inflammation with possible granulomatous lesions.* Phaeoacremonium parasiticum* grew only from the bronchoalveolar lavage culture but not from the biopsy culture. The patient improved after the administration of voriconazole and caspofungin [21, 26].

*Phaeoacremonium* species are typically isolated from thorns, wood, and soil. Human infection can be caused by traumatic implantation or occurs in the setting of immunocompromising conditions. Twenty-seven cases of human infections with* Phaeoacremonium* species have been reported in the literature. In immunocompetent hosts,* Phaeoacremonium* has been reported to cause subcutaneous phaeohyphomycosis, osteomyelitis, endophthalmitis, and onychomycosis. Successful outcomes have been achieved with debridement and antifungals (Table 1). In immunocompromised patients,* Phaeoacremonium* causes more severe disease and has been reported to cause endocarditis, brain abscesses, cavitary lung nodules, and disseminated infections. Disseminated infections in severely immunocompromised hosts are associated with poor outcomes and death (Table 2).

The other dematiaceous fungus isolated from this patient was* Dactylaria constricta*. It grew only from culture of bronchial fluid and not from the biopsy.* Mycobacterium avium-intracellulare* complex (MAC) also grew only from the culture of bronchial fluid. Given our patient's exposure history (gardening, exposure to sheds and barns) and no growth of these organisms from the biopsy specimen, it is likely that these organisms were merely colonizers of her respiratory tract and not pathogens. Moreover, resolution of her illness without treatment for MAC suggests that it was not a pathogen.

There is no standard antifungal regimen described for* Phaeoacremonium* in the literature. Posaconazole is the most recently approved triazole with an extended spectrum of activity against a wide variety of fungi. Posaconazole was chosen over other azoles because it is well tolerated and has a favorable side effect profile and a low potential of drug interactions compared to other azoles. Posaconazole inhibits the metabolism of calcineurin inhibitors. Failure to adjust tacrolimus dosing can result in supratherapeutic levels of tacrolimus and harm the kidney [28]. Our patient responded well to the treatment with no relapse of infection during 4 years of follow-up. Her kidney allograft continues to function well, with creatinine levels ranging between 1 and 1.3 mg/dL.

## Figures and Tables

**Table 1:** Skin and subcutaneous infection, osteomyelitis, endophthalmitis, and onychomycosis due to *Phaeoacremonium *species.

Number	Reference	Species	Age/sex	Underlying condition	Clinical disease	Treatment	Outcome
1	Padhye et al., 1998 [4]	*P*. *inflatipes *	83/F	None	Subcutaneous infection of the foot	Excision	Complete healing
2	Matsui et al., 1999 [5]	*P*. *rubrigenum *	61/F	Rheumatoid arthritis	Subcutaneous mass of the foot	Excision Itraconazole Fluconazole	Recurred
3	Kitamura et al., 2000 [6]	*P*. *parasiticum *	59/F	None	Subcutaneous nodule below the knee	Excision	Complete healing
4	Guarro et al., 2003 [7]	*P*. *aleophilum *	19/M	None	Fistulized nodule on the ankle	Excision (six times) Itraconazole	Cured
5	Guarro et al., 2003 [7]	*P*. *rubrigenum *	55/M	Renal transplant	Multiple nodules of ankle and foot	Excision Itraconazole Terbinafine Fluconazole	Not resolved
6	Llinas et al., 2005 [8]	*Phaeoacremonium *species	54/F	Myelodysplastic syndrome, IgA deficiency	Olecranon bursitis	Excision Itraconazole	Resolved
7	Baddley et al., 2006.[9]	*P*. *parasiticum *	40/M	Cardiac transplant	Multiple skin lesions	Amphotericin B, Itraconazole Debridement	Died
8	Hemashettar et al., 2006 [10]	*P*. *krajdenii *	41/M	None	Mycetoma	Itraconazole Debridement	Recurred
9	Marques et al., 2006 [11]	*P*. *parasiticum *	49/M	Renal transplant	Draining cystic tumors on the foot	Itraconazole Amphotericin B	Improved
10	Huynh et al., 2007 [12]	*P*. *parasiticum *	19/M	Penetrating globe injury	Endophthalmitis	Amphotericin B Voriconazole	Improved
11	Farina et al., 2007 [13]	*P*. *parasiticum *	41/M	Kidney transplant	Subcutaneous nodule on the forefinger	Excision	Resolved
12	Baradkar et al., 2009 [14]	*P*. *parasiticum *	26/F	None	Subcutaneous abscess on the forearm	Debridement Amphotericin B Itraconazole	Resolved
13	Sun et al., 2011 [15]	*P*. *parasiticum *	55/M	None	Onychomycosis	Diseased nail was trimmed off Topical sulconazole	Cured
14	Aguilar et al., 2011.[16]	*P*. *parasiticum *	52/F	Type 2 diabetes hypothyroidism.	Eumycetoma	Surgery (multiple times), Itraconazole	Improved
15	Baradkar et al., 2011 [17]	*P*. *infalitipes *	30/M	None	Subcutaneous mass of the foot	Debridement, Amphotericin B, Itraconazole	Cured
16	Choi et al., 2011 [18]	*Phaeoacremonium *species	54/M	Renal transplant	Subcutaneous mass on the third finger	Excision	Resolved
17	Mazzurco et al., 2012 [19]	*Phaeoacremonium *species	74/M	Rheumatoid arthritis, on infliximab	Nodule on the leg	Excision Itraconazole	Resolved
18	Furudate et al., 2012 [20]	*P*. *rubrigenum *	76/F	Still's disease, on prednisolone	Subcutaneous nodules on the leg	Debridement, Itraconazole	Resolved
19	To et al., 2012 [21]	*P*. *parasiticum *	69/M	Diabetes mellitus	Right knee Pain and swelling	Arthrotomy and drainage, Itraconazole, total knee replacement	Improved
20	Guarro et al., 2006 [22]	*P*. *venezuelense *	28/M	Chronic myeloid leukemia	Subcutaneous mycoses	Surgical excision	Not known

**Table 2:** Invasive and disseminated infections due to *Phaeoacremonium *species.

Number	Reference	Species	Age/sex	Underlying condition	Clinical disease	Treatment	Outcome
21	Heath et al., 1997 [23]	*P*. *parasiticum *	45/M	Liver transplant	Infective endocarditis, fungemia, and skin lesion	Amphotericin B, fluconazole	Died
22	Wang et al., 2005 [24]	*P*. *inflatipes *	18- month boy	Aplastic anemia	Fungemia	Amphotericin B	Died
23	Baddley et al., 2006 [9]	*P*. *parasiticum *	31/F	Aplastic anemia	Fungemia, skin lesions	Amphotericin B	Died
24	McNeil et al., 2011 [25]	*P*. *parasiticum *	24/M	Chronic granulomatous disease, end-stage kidney disease	Brain abscess	Amphotericin B, voriconazole, caspofungin	Died
25	To et al., 2012 [21]	*P*. *parasiticum *	26/M	Renal transplant	Cavitary lesion of lung	Voriconazole Caspofungin	Responded, but died
26	Shah et al., 2013 [26]	*P*. *parasiticum *	74/M	Lung transplant	Lung nodules of native lung	Caspofungin Voriconazole	Improved
27	Larbcharoensub et al., 2013 [27]	*Scedosporium apiospermum *and *P*. *parasiticum *	49 y/o	Renal transplant	Multiple brain abscesses	Voriconazole	Improved
28	Present case	*Phaeoacremonium *species	49/F	Renal transplant	Lung nodules	Posaconazole	Improved
